# Dual SGLT1/2 inhibition with sotagliflozin: a pharmaceutical breakthrough in heart failure management and cardiometabolic risk reduction

**DOI:** 10.3389/fmed.2025.1733918

**Published:** 2026-01-02

**Authors:** Ghaith K. Mansour, Sarah B. Hammo, Muhammad Raihan Sajid

**Affiliations:** 1 Department of Pharmaceutical Sciences, College of Pharmacy, Alfaisal University, Riyadh, Saudi Arabia; 2 Jordan University Hospital, The University of Jordan, Amman, Jordan; 3 Department of Pathology, College of Medicine, Alfaisal University, Riyadh, Saudi Arabia

**Keywords:** cardiovascular outcomes, diabetes, dual inhibition, heart failure, SGLT inhibitor, sotagliflozin

## Abstract

Heart failure remains a global health crisis with high morbidity and mortality. Sotagliflozin, a first-in-class dual sodium-glucose cotransporter 1 and 2 (SGLT1/2) inhibitor, offers a novel therapeutic approach. Its dual mechanism concurrently inhibits renal (SGLT2) and intestinal (SGLT1) glucose transporters, enhancing glycemic control and providing additive benefits in cardiovascular risk reduction, blood pressure, and body weight management. Recent randomized trials demonstrate that sotagliflozin significantly lowers the risk of major cardiovascular events, heart failure hospitalizations, and all-cause mortality in patients with and without diabetes. While its safety profile is generally favorable, it necessitates monitoring for gastrointestinal effects and diabetic ketoacidosis. This review synthesizes mechanistic insights, clinical evidence, and practical considerations for integrating sotagliflozin into heart failure management, positioning it as a promising innovation in cardiometabolic therapeutics.

## Introduction and background on sotagliflozin

The management of heart failure (HF) and cardiometabolic disease has been transformed by sodium-glucose cotransporter (SGLT) inhibitors. While selective SGLT2 inhibitors have established benefits, sotagliflozin represents a significant pharmacological advancement as the first dual SGLT1/2 inhibitor approved for cardiovascular indications. This review aims to critically appraise the unique value of dual SGLT1/2 inhibition, synthesizing evidence on its mechanisms, clinical efficacy, safety, and place in therapy, particularly for patients with heart failure.

Unlike traditional SGLT2-selective inhibitors, sotagliflozin was specifically designed to target both renal glucose reabsorption (via SGLT2) and intestinal glucose absorption (via SGLT1) (1). This dual action provides incremental benefits by mitigating postprandial hyperglycemia and offering a broader mechanism of cardiometabolic protection (2). Initially developed for diabetes, its most significant regulatory milestone came in 2023 when it became the first SGLT inhibitor to receive Food and Drug Administration (FDA) approval for heart failure across the entire spectrum of ejection fraction (3). This approval addressed a critical unmet need, underscoring the importance of effective cardiovascular medications in an era where cardiometabolic diseases like diabetes affect hundreds of millions globally (4). The drug’s unique profile, with approximately 20-fold higher inhibitory activity for SGLT2 over SGLT1, allows for effective renal glucose handling while providing meaningful intestinal inhibition (5).

### Heart failure and its pathophysiology

Heart failure is a complex clinical syndrome resulting from structural or functional cardiac impairment, leading to symptoms like breathlessness, fatigue, and fluid retention (6, 7). It is classified by left ventricular ejection fraction (LVEF) into HF with reduced (HFrEF), mildly reduced (HFmrEF), and preserved (HFpEF) ejection fraction (7).

The pathophysiology involves maladaptive neurohormonal activation (e.g., RAAS and sympathetic nervous system) and cardiac remodeling, which initially compensate for reduced cardiac output but ultimately exacerbate disease progression (6, 8). Key processes relevant to SGLT inhibitor mechanisms include:

- Cardiac remodeling: chronic stress leads to pathological changes in the heart’s size, shape, and function, impairing contractility.
- Energetic deficits: mitochondrial dysfunction and oxidative stress reduce ATP production, compromising the energy-demanding processes of cardiac contraction and relaxation (6, 9).
- Systemic Congestion: A hallmark of HF, congestion manifests as pulmonary edema (due to fluid redistribution) and peripheral edema (due to fluid retention) (10).

Despite continuous advances in therapeutics, heart failure remains a major burden on healthcare systems with high incidence of morbidity and mortality, with up to 75% mortality at five years. The mechanistic insights revealed by current research offer novel therapeutic approaches and targets that are gaining traction towards translation in patients. Understanding these pathophysiological mechanisms is essential for developing targeted interventions and improving patient outcomes, as the suppression of lusitropy, S-glutathionylation of sarcomere proteins, connexin 43 phosphorylation, alterations in cytoskeletal and calcium cycling regulators, and transcriptional heterogeneity in cardiomyocytes represent key molecular mechanisms implicated in cardiomyopathies (11). Understanding these pathways is crucial, as SGLT inhibitors like sotagliflozin are thought to exert benefits by improving myocardial energetics, reducing remodeling, and promoting diuresis (6, 12).

### Chemistry and formulation of sotagliflozin

Sotagliflozin is chemically designated as (2S,3R,4R,5S,6R)-2-(4-chloro-3-(4-ethoxybenzyl)phenyl)-6-(methylthio)tetrahydro-2H-pyran-3,4,5-triol. Its molecular formula is C₂₁H₂₅ClO₅S, corresponding to a molecular weight of 424.94 g/mol. The compound appears as a white to off-white solid and exhibits negligible solubility in water. Each film-coated tablet delivers either 200 mg or 400 mg of the active pharmaceutical ingredient, sotagliflozin. The tablet core formulation includes excipients such as colloidal silicon dioxide, croscarmellose sodium, magnesium stearate, microcrystalline cellulose, and talc. The composition of the film coating varies by dosage strength. For the 200 mg tablet, the coating contains indigo carmine aluminum lake, polyethylene glycol, partially hydrolyzed polyvinyl alcohol, talc, and titanium dioxide. In contrast, the 400 mg tablet film coating comprises hypromellose, lactose monohydrate, titanium dioxide, triacetin, and yellow iron oxide. The black imprint ink used on both strengths includes ammonium hydroxide, black iron oxide, isopropyl alcohol, N-butyl alcohol, propylene glycol, and shellac (3).

Sotagliflozin represents a sophisticated chemical entity specifically designed to achieve dual SGLT1/2 inhibition through its unique molecular architecture. The drug’s chemical design incorporates structural features that allow for differential binding affinity to both SGLT1 and SGLT2 transporters, with the 20-fold selectivity for SGLT2 being achieved through specific molecular modifications. The discovery that intestinal SGLT-1 inhibition can provide a novel opportunity to control hyperglycemia, through a multifactorial mechanism, encouraged the design of compounds like sotagliflozin that could selectively target both transporters. The molecular structure of sotagliflozin has been optimized through extensive structure–activity relationship studies that focused on achieving the optimal balance between SGLT1 and SGLT2 inhibition. The compound’s design reflects advances in understanding the structural requirements for dual inhibition, incorporating features that allow for effective binding to both transporter subtypes while maintaining drug-like properties suitable for oral administration (13).

The pharmaceutical development of sotagliflozin involved extensive formulation optimization to ensure adequate bioavailability and stability for clinical use. The drug is formulated as oral tablets designed to provide consistent pharmacokinetic profiles across different patient populations. The formulation has been designed to support both the 200 mg and 400 mg dosing regimens that have been evaluated in clinical trials. Clinical pharmacokinetic studies have demonstrated that sotagliflozin exhibits predictable absorption characteristics following oral administration, with the drug being well-tolerated across different dosing regimens. The pharmaceutical formulation has been optimized to ensure consistent bioavailability and to minimize potential food effects on absorption (5).

### Sotagliflozin’s pharmacological effects and mechanism of action

**Figure 1:**
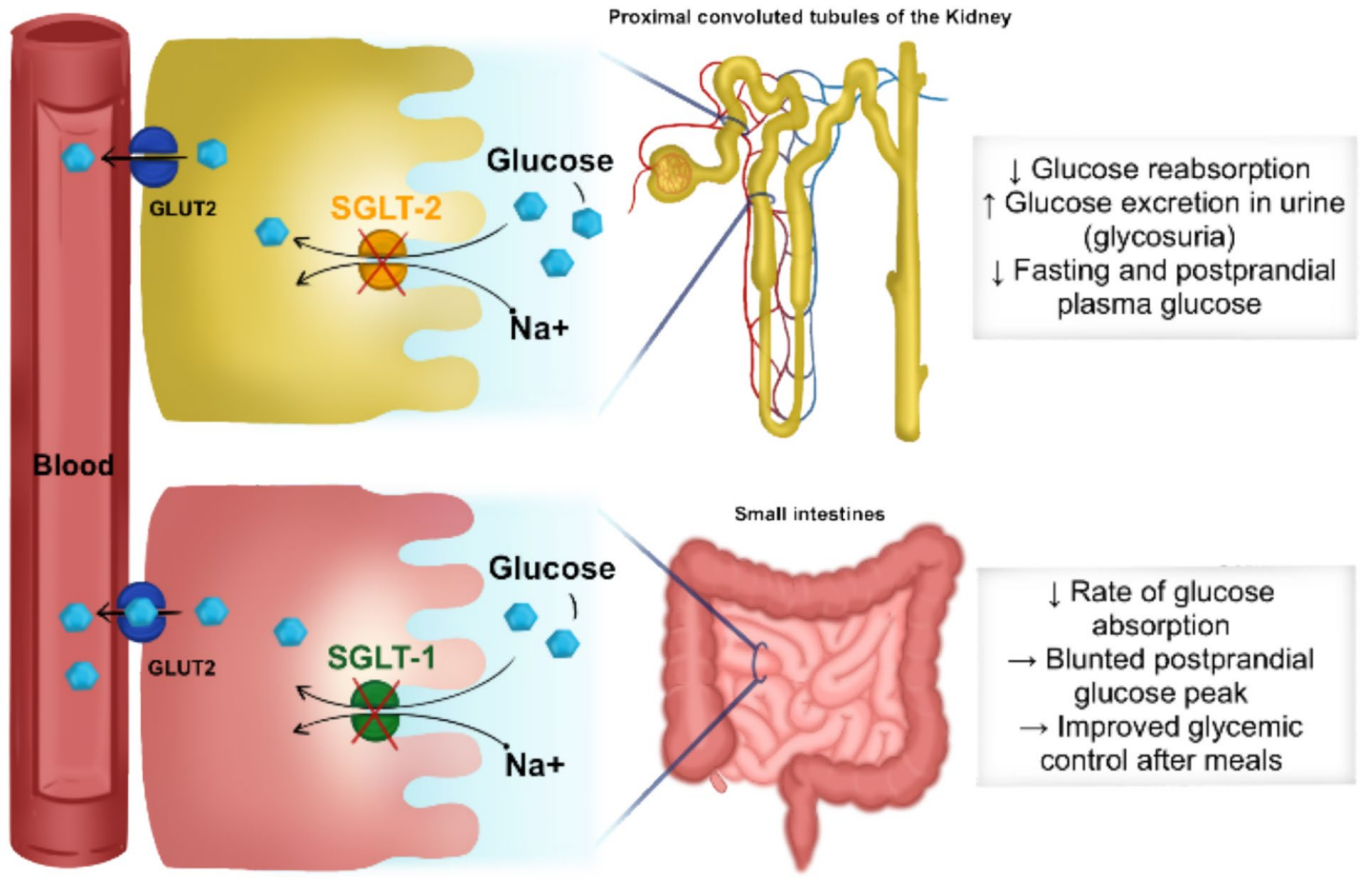
Sotagliflozin a dual SGLT-1 and SGLT-2 inhibitor.

Sotagliflozin’s pharmacological effects stem from its unique ability to inhibit both SGLT1 and SGLT2 transporters with differential selectivity, exhibiting approximately 20-fold higher selectivity for SGLT2 compared to SGLT1 (2). This dual inhibition mechanism represents a significant advancement over traditional SGLT2-selective inhibitors, as it targets glucose homeostasis through multiple pathways simultaneously. The drug’s mechanism involves blocking SGLT1 in the gastrointestinal tract, which contributes to glucose reabsorption, while simultaneously inhibiting SGLT2 in the proximal renal tubule (8) (Figure 1**)**. The differential inhibition profile allows sotagliflozin to provide comprehensive glucose control by targeting both dietary glucose absorption and renal glucose reabsorption. Studies have demonstrated that sotagliflozin decreases postprandial glucose concentrations by delaying intestinal glucose absorption, an effect that is mediated through its SGLT1 inhibitory activity. This mechanism provides additional glycemic control beyond what can be achieved through SGLT2 inhibition alone (6).

The cardiovascular effects of sotagliflozin extend beyond its glucose-lowering properties, demonstrating significant benefits in heart failure outcomes across different patient populations (7). Preclinical studies have shown that sotagliflozin improves cardiac outcomes in normoglycemic mouse models of cardiac pressure overload, suggesting that its cardiovascular benefits are independent of glucose-lowering effects (11). The drug’s ability to attenuate cardiac dysfunction and remodeling in myocardial infarction models further supports its cardioprotective mechanisms (49). Animal studies have demonstrated that sotagliflozin treatment results in improved cardiac function and reduced infarct size compared with vehicle-treated groups (49). Additionally, sotagliflozin improved cardiac remodeling as shown by decreased cardiac hypertrophy and cardiac apoptosis in post-myocardial infarction hearts. These preclinical findings provide mechanistic support for the clinical cardiovascular benefits observed in human trials (14).

The metabolic effects of sotagliflozin encompass multiple pathways beyond glucose homeostasis, including effects on body weight, blood pressure, and lipid metabolism. In early clinical studies, sotagliflozin demonstrated significant reductions in body weight, with patients experiencing a 1.7 kg decrease compared to a 0.5 kg gain in placebo groups. The drug’s effects on metabolic parameters extend to improvements in glycemic variability and time in target glucose ranges. Studies have shown that sotagliflozin reduces the percentage of time spent in hyperglycemic ranges while improving overall glycemic control. The drug’s unique mechanism allows for complementary effects on both fasting and postprandial glucose concentrations, providing comprehensive glucose management through its dual inhibitory profile (10).

### Reno-protective mechanisms of dual SGLT1/2 inhibition

The reno-protective benefits of sotagliflozin extend beyond the established class effects of SGLT2 inhibition. While SGLT2 inhibition mitigates glomerular hyperfiltration by restoring tubuloglomerular feedback, leading to reduced intraglomerular pressure and albuminuria, dual SGLT1/2 inhibition offers additional pathways for protection. By inhibiting SGLT1 in the proximal intestine, sotagliflozin reduces postprandial hyperglycemia (15, 16). This sustained improvement in glycemic variability lessens the glucose-mediated oxidative stress and inflammatory cytokine release that contributes to renal endothelial dysfunction and fibrosis. Intestinal SGLT1 inhibition delays glucose absorption and promotes the release of glucagon-like peptide-1 (GLP-1) and peptide YY (PYY) (9). These incretin hormones have been shown to exert anti-inflammatory and anti-fibrotic effects directly on the kidney, potentially providing an additive reno-protective signal alongside the direct renal effects of SGLT2 inhibition. The combined natriuresis and osmotic diuresis from dual inhibition promote sustained plasma volume reduction and blood pressure control, reducing mechanical stress on the glomeruli (16–19).

### Pharmacokinetics, pharmacodynamics, and pharmacogenomics of sotagliflozin

Sotagliflozin demonstrated predictable, dose-proportional pharmacokinetics across multiple ascending-dose studies. Sotagliflozin displayed linear pharmacokinetics at 200 mg and 400 mg doses once daily for 8 days in Chinese healthy subjects. The compound has proved to be orally bioavailable with reproducible pharmacokinetics following oral administration. PK of sotagliflozin and the principal metabolite LX4211-GLU have been well-defined with extensive collection blood samples taken pre- and post-dose over the course of multiple-dose administration. The drug shows predictable elimination with no clinically of dose accumulation upon repeated once daily administration. These pharmacokinetic characteristics are compatible with a once a day dosing and a preferred design for clinical studies (19). The glucose-dependent and glucose-independent mechanisms of sotagliflozin mediate the pharmacodynamic effects that contribute to its clinical action. Studies have shown that sotagliflozin, with its dual SGLT1/2 inhibition, reduces glucose in a dose-dependent manner. The pharmacodynamic profile of the drug includes impacts on urinary glucose excretion, corresponding to its SGLT2 inhibitory activity as well as published effects in postprandial glucose absorption (mediated via SGLT1 inhibition) and other measures such as insulin sensitivity. Interesting mechanistic evaluation studies with PBPK modeling have also been carried out to elucidate the specific inhibition of SGLT1 and 2 transporters by sotagliflozin. Such studies have lent valuable insights into the possibility for treatment with this drug at doses that could reasonably be anticipated to achieve a clinically meaningful extent of inhibition of both transporter subtypes. Modeling has been used to relate drug exposure with effect, a so-called pharmacodynamic model (20). Although prior pharmacogenomic studies of sotagliflozin are not available in the current literature, the metabolic pathways and excretion characteristics of the drug support pharmacogenomic variability. The production of the major metabolite LX4211-GLU suggests a role for drug-metabolizing enzymes possibly subject to genetic polymorphisms. Safety and efficacy of sotagliflozin are heavily influenced by pharmacogenomic factors, which severely limits broader clinical applicability, and understanding these factors will be an important area for future investigation. Although population pharmacokinetic analyses in clinical trials have elucidated some potential factors impacting sotagliflozin exposure, well-controlled pharmacogenomics studies are required to completely define these associations. The reproducible pharmacokinetics of the drug across populations indicate that large pharmacogenomic effects are unlikely, but confirmatory findings will be important (19).

### Comparative profile: dual SGLT1/2 vs. selective SGLT2 inhibition

While both sotagliflozin and selective SGLT2 inhibitors (e.g., empagliflozin, dapagliflozin) provide foundational cardiorenal benefits, their distinct mechanisms translate into differences in clinical effects (summarized in Table 1) (12, 21). The principal differentiator is the additional inhibition of intestinal SGLT1 by sotagliflozin. This dual action offers more significant reductions in postprandial glucose excursions, which may be particularly advantageous for patients with high carbohydrate intake or poor postprandial control. Furthermore, the SGLT1-mediated delay in glucose absorption and subsequent incretin release (GLP-1) represents a unique, non-renal pathway for systemic metabolic and potential anti-inflammatory benefits (9, 17). In contrast, selective SGLT2 inhibitors have a more pronounced effect on fasting plasma glucose due to potent, isolated blockade of renal glucose reabsorption. The clinical significance of these mechanistic differences in long-term hard outcomes is an active area of investigation.

**Table 1:** Comparative profile of sotagliflozin and selective SGLT2 inhibitors.

Feature	Sotagliflozin (dual SGLT1/2 inhibitor)	Selective SGLT2 inhibitors (e.g., empagliflozin, dapagliflozin) (24)
Primary mechanism	Inhibits SGLT2 (Kidney) + SGLT1 (Intestine)	Inhibits SGLT2 (Kidney)
Glycemic control	Reduces both fasting and postprandial glucose	Primarily reduces fasting plasma glucose
Unique metabolic effects	Delays intestinal glucose absorption; promotes GLP-1 release	Minimal direct intestinal effects
Cardiovascular benefits	Robust reduction in HF events across all EF spectra; supported by dedicated trials in hospitalized patients (SOLOIST-WHF)	Robust reduction in HF events; extensive outcome data in stable outpatient populations (e.g., EMPEROR-Preserved, DAPA-HF)
Renal effects	Potential additive benefit via gut-kidney axis and improved postprandial control; efficacy shown in CKD population (SCORED)	Well-established renoprotection in dedicated trials (e.g., DAPA-CKD, EMPA-KIDNEY)
Key safety consideration	Higher incidence of diarrhea (SGLT1-mediated)	Lower risk of diarrhea

### Clinical trials and efficacy of sotagliflozin

The SOLOIST-WHF (Sotagliflozin in Patients with Diabetes and Recent Worsening Heart Failure) trial represents one of the most significant clinical investigations of sotagliflozin’s cardiovascular effects. This landmark trial studied the effects of sotagliflozin in patients with type 2 diabetes who had been recently hospitalized for worsening heart failure. The study demonstrated significant cardiovascular benefits with sotagliflozin treatment, establishing its role in heart failure management.

The SOLOIST-WHF trial provided compelling evidence for the initiation of SGLT2 inhibitors in patients hospitalized with worsening heart failure, supporting the concept that these medications should be started during hospitalization rather than waiting for outpatient initiation. The trial results contributed to updated meta-analyses showing the broad benefits of sodium-glucose co-transporter 2 inhibitors across different heart failure populations (22). The study enrolled patients across the spectrum of left ventricular ejection fraction (including HFrEF and HFpEF). The primary endpoint was the total number of deaths from cardiovascular causes and hospitalizations and urgent visits for heart failure. Sotagliflozin demonstrated a significant 51% reduction in the relative risk of the primary endpoint compared to placebo. The study demonstrated significant cardiovascular benefits with sotagliflozin treatment, establishing its role in heart failure management (22).

The SCORED (Sotagliflozin on Cardiovascular and Renal Events in Patients with Type 2 Diabetes and Moderate Renal Impairment Who Are at Cardiovascular Risk) trial focused on patients with type 2 diabetes and chronic kidney disease, evaluating sotagliflozin’s effects on cardiovascular and renal outcomes. The study enrolled patients with diabetes mellitus (hemoglobin A1c ≥ 7%) and CKD (eGFR, 25–60 mL/min per 1.73 m^2^ with or without albuminuria) who were randomized 1:1 to sotagliflozin 200 mg/d and uptitrated to 400 mg/d versus placebo. The primary endpoint was a composite of cardiovascular deaths, hospitalizations for heart failure, and urgent visits for heart failure. Sotagliflozin significantly reduced the relative risk of the primary endpoint by 26% compared to placebo (23). The SCORED trial’s design and implementation provided important insights into the use of dual SGLT1/SGLT2 agents in patients with chronic kidney disease. The trial contributed to understanding sotagliflozin’s 20:1 SGLT2:SGLT1 receptor affinity and its implications for cardiovascular risk reduction in patients with diabetes and kidney disease (23).

The inTandem program represents a comprehensive clinical development program for sotagliflozin in type 1 diabetes, encompassing multiple phase 3 trials that evaluated the drug’s efficacy and safety in this population. The inTandem1 and inTandem2 trials randomized adults with type 1 diabetes on optimized insulin to sotagliflozin 200 mg, 400 mg, or placebo. The primary endpoint was changes in HbA1c at 24 weeks. Both sotagliflozin doses demonstrated significant HbA1c reductions, weight loss, and lower insulin doses, but with an increased risk of diabetic ketoacidosis. The program demonstrated improvements in time in range and glycemic variability with sotagliflozin in combination with insulin in adults with type 1 diabetes. Pooled analyses of 24-week continuous glucose monitoring data from the inTandem program provided comprehensive evidence of sotagliflozin’s glycemic benefits (24). The inTandem4 trial specifically evaluated dose-dependent glycometabolic effects of sotagliflozin on type 1 diabetes over 12 weeks, demonstrating the drug’s ability to provide sustained glycemic improvements. These studies established sotagliflozin’s efficacy in type 1 diabetes management, though regulatory approval for this indication has faced challenges related to safety considerations (25).

Multiple meta-analyses have synthesized the evidence for sotagliflozin across different clinical conditions, providing comprehensive assessments of its efficacy and safety profile. A comprehensive meta-analysis of sotagliflozin as a dual sodium-glucose-cotransporter 1/2 inhibitor for heart failure in type 2 diabetes demonstrated consistent benefits across multiple endpoints. These analyses have evaluated randomized controlled trials of sotagliflozin in type 2 diabetes, focusing on heart failure and cardiovascular outcomes (12). Bayesian network meta-analyses have compared sotagliflozin with other cardiovascular interventions, demonstrating its competitive efficacy profile. Studies comparing sotagliflozin with placebo, dapagliflozin, and empagliflozin in adult T2DM patients with heart failure or cardiovascular risks have shown favorable outcomes for sotagliflozin across multiple endpoints (26) (see Table 2).

**Table 2:** Key clinical trials of sotagliflozin.

Trial (acronym)	Patient population	Intervention	Primary endpoint and key findings	Clinical significance
SOLOIST-WHF (22)	T2D recently hospitalized for worsening HF	Sotagliflozin 200 mg, uptitrated to 400 mg once daily vs. Placebo	Primary: Total number of CV deaths, HF hospitalizations, and urgent HF visits. 51% relative risk reduction vs. placebo.	Supported in-hospital initiation of SGLT inhibitors in worsening HF, demonstrating benefit across EF spectrum.
SCORED (23)	T2D with moderate CKD (eGFR 25–60)	Sotagliflozin 200 mg, uptitrated to 400 mg once daily vs. Placebo	Primary: Composite of CV deaths, HF hospitalizations, and urgent HF visits. 26% relative risk reduction vs. placebo.	Demonstrated efficacy in a high-risk population with T2D and CKD, reducing heart failure events.
inTandem Program (24, 25)	T1D on optimized insulin therapy	Sotagliflozin 200 mg or 400 mg once daily vs. Placebo	Primary: change in HbA1c at 24 weeks. Significant HbA1c reduction, weight loss, and lower insulin dose vs. placebo. Increased risk of diabetic ketoacidosis.	Established efficacy as an adjunct in T1D, though DKA risk limited regulatory approval.

### Cost-effectiveness and pharmacoeconomics

Comprehensive cost-effectiveness analyses have evaluated sotagliflozin’s economic value across different clinical populations in the United States healthcare system. A detailed cost-effectiveness analysis model for sotagliflozin compared with insulin monotherapy for patients with type 1 diabetes and chronic kidney disease demonstrated favorable economic outcomes. The analysis employed a Markov model from a US payer perspective, evaluating clinical and economic outcomes over 30 years.

The cost-effectiveness model demonstrated that sotagliflozin add-on therapy improved survival, extending life expectancy by 1.27 years (13.08 with sotagliflozin vs. 11.81 with insulin monotherapy). During the first 10 years after treatment initiation, dialysis and transplant utilization decreased by 3.06 and 1.73 per 1,000 patients, respectively. Quality-adjusted life years (QALYs) per patient increased by 0.63, with an incremental cost-effectiveness ratio (ICER) of \$115,677 per QALY, falling below the \$150,000/QALY willingness-to-pay threshold in 59% of probabilistic sensitivity analysis simulations (27). Budget impact analyses have assessed the financial implications of sotagliflozin adoption from various payer perspectives. Economic impact studies of sotagliflozin among patients with heart failure and type 2 diabetes from the US payer perspective have demonstrated that while sotagliflozin increases pharmacy costs for recently hospitalized heart failure patients with type 2 diabetes, approximately 21–68% of pharmacy costs were offset from reduced rehospitalization and emergency department visits. The budget impact analysis showed that for all-payer plans, annual per-user costs increased by \$4,996 because of higher pharmacy costs, but this was partially offset by reduced healthcare utilization. The budget impact of sotagliflozin was estimated at \$0.08 per member per month (PMPM) in year 1 and \$0.38 in year five, corresponding with total plan costs of \$75,736 in year 1 and \$378,681 by year 5 (28).

Comparative economic analyses have evaluated sotagliflozin against other SGLT2 inhibitors and standard therapies. A cost per outcome analysis comparing sotagliflozin versus dapagliflozin to improve outcomes in patients with diabetes and worsening heart failure demonstrated sotagliflozin’s potential cost advantages. The analysis calculated that preventing one cardiovascular event required treating 20 patients with sotagliflozin, as opposed to 23 for dapagliflozin (29). Cost-effectiveness analyses have consistently demonstrated that sotagliflozin provides clinical benefits that justify its cost across different healthcare systems. Studies evaluating sotagliflozin for the treatment of patients with diabetes and recent worsening heart failure have shown that the drug decreased annualized rehospitalization rates by 34.5%, annualized emergency department visits by 40.0%, and annualized mortality by 18.0% relative to standard of care (30). Economic evaluations of sotagliflozin have been conducted across multiple healthcare systems internationally, providing insights into its cost-effectiveness across different economic environments. Studies examining how adoption of sotagliflozin can impact US health system reimbursement under alternative payment models have demonstrated that although sotagliflozin adoption reduced health system revenue in fee-for-service payment models, it led to a net positive financial impact after accounting for alternative payment model bonus payments. The economic impact analysis showed that sotagliflozin adoption resulted in savings of \$1,200 per person for the Bundled Payments for Care Improvement-Advanced program, and \$1,078 per person for Accountable Care Organizations. Based on the national average composition of alternative payment model reimbursement, sotagliflozin adoption resulted in a \$1,576 increase in margin per patient with heart failure (31).

Pharmacoeconomic analyses indicate that sotagliflozin is a cost-effective intervention. While the drug acquisition cost is higher than standard care, this is largely offset by a significant reduction in costs associated with heart failure hospitalizations and other cardiovascular events. Studies from the US healthcare perspective have shown that the clinical benefits---including reduced mortality and hospitalization rates---translate into improved economic outcomes, with acceptable incremental cost-effectiveness ratios. The budget impact is moderated by savings from decreased healthcare utilization, making sotagliflozin a valuable addition to formularies for managing heart failure and related cardiometabolic conditions (27–31).

### Toxicological and safety profile of sotagliflozin

Sotagliflozin’s safety profile is generally favorable and consistent with the SGLT inhibitor class, but it has unique considerations. Preclinical studies in various animal models have shown no unexpected toxicities (32–34). Clinically, the most common adverse events are genital mycotic infections (a class effect) and diarrhea, the latter being linked to its SGLT1 inhibitory activity in the gut (26). Volume depletion-related events, such as hypotension, have also been reported at a higher incidence compared to placebo (26).

The most significant safety concern is the risk of diabetic ketoacidosis (DKA), particularly in patients with type 1 diabetes. This risk was a primary reason for the FDA’s decision to withhold approval for the type 1 diabetes indication (3). While meta-analyses have not shown a significantly increased DKA risk compared to other SGLT2 inhibitors in type 2 diabetes, careful patient selection, education, and monitoring are imperative (26, 35). This safety controversy underscores the need for a balanced approach when prescribing sotagliflozin.

### Adverse events and long-term outcomes of sotagliflozin

The adverse event profile of sotagliflozin has been characterized through extensive clinical trial data and post-marketing surveillance (3). The most commonly reported adverse events associated with sotagliflozin treatment include gastrointestinal effects, particularly diarrhea, which has been observed more frequently compared to placebo in clinical trials (26). Genital infections represent another category of adverse events that have been observed with sotagliflozin treatment, consistent with the class effect of SGLT inhibitors (3).

Meta-analyses have provided quantitative assessments of adverse event frequencies with sotagliflozin treatment across different patient populations. These analyses have shown that while sotagliflozin is generally well-tolerated, certain adverse events occur with greater frequency compared to placebo, requiring appropriate patient counseling and monitoring. The gastrointestinal adverse events, particularly diarrhea, appear to be related to the drug’s SGLT1 inhibitory activity in the intestine (26). Serious adverse events associated with sotagliflozin treatment have been systematically evaluated through clinical trials and safety analyses (35). Diabetic ketoacidosis represents the most significant serious adverse event of concern with sotagliflozin, particularly in patients with type 1 diabetes. This risk has been a major factor in regulatory decision-making, leading to the FDA’s withholding of approval for type 1 diabetes indications (3).

Safety meta-analyses have shown that sotagliflozin exhibited no increased risk for diabetic ketoacidosis compared to other SGLT2 inhibitors when used in appropriate patient populations. However, the risk of diabetic ketoacidosis remains a clinical consideration that requires appropriate patient selection and monitoring. Healthcare providers must be aware of the risk factors and clinical presentation of diabetic ketoacidosis when prescribing sotagliflozin (26). Long-term safety data for sotagliflozin continue to be collected through extended follow-up studies and real-world evidence generation. The available long-term safety data suggest that sotagliflozin maintains its favorable safety profile with continued use, though longer-term studies are needed to fully characterize its safety across extended treatment periods. Cardiovascular safety outcomes from major clinical trials have demonstrated that sotagliflozin does not increase cardiovascular risk and may provide cardiovascular benefits (35). The long-term safety profile of sotagliflozin appears consistent with its mechanism of action and the known safety profile of the SGLT inhibitor class. Continued pharmacovigilance and long-term safety monitoring remain important for fully characterizing the drug’s benefit–risk profile in clinical practice. Real-world evidence studies will be crucial for understanding sotagliflozin’s safety profile in broader patient populations (35).

### Patient-reported outcomes

Patient-reported outcomes represent an important dimension of sotagliflozin’s clinical benefit profile, complementing traditional clinical endpoints with assessments of how the drug affects patients’ daily lives and well-being. Studies from the inTandem program have evaluated patient-reported outcomes in adults with type 1 diabetes treated with sotagliflozin, focusing on improvements in glycemic control and quality of life measures. These assessments have shown that improvements in glycemic variability and time in range translate to meaningful benefits from the patient perspective. The relationship between sotagliflozin’s clinical effects and patient-reported outcomes has been evaluated through validated quality of life instruments that capture both diabetes-specific and general health-related quality of life measures. Patients treated with sotagliflozin have reported improvements in diabetes-related quality of life measures, particularly related to better glycemic control and reduced glucose excursions (24).

Patient-reported symptom assessments have provided insights into how sotagliflozin affects various aspects of disease-related symptoms and functional capacity. Studies have evaluated the effects of sotagliflozin on clinical markers associated with cardiorenal protection through exploratory analyses that include patient-reported functional outcomes. These assessments have contributed to understanding how sotagliflozin’s physiological effects translate to improvements in patient-experienced symptoms. The functional outcomes associated with sotagliflozin treatment extend beyond glucose control to include cardiovascular-related symptoms and overall functional capacity. Patients with heart failure treated with sotagliflozin have reported improvements in symptoms related to cardiovascular function, consistent with the drug’s demonstrated cardiovascular benefits (36). Treatment satisfaction with sotagliflozin has been evaluated through patient-reported outcome measures that assess various aspects of the treatment experience. The once-daily dosing regimen and oral formulation of sotagliflozin contribute to treatment convenience and patient acceptance. Patient feedback regarding sotagliflozin treatment has generally been positive, with patients appreciating the glycemic benefits and relative tolerability of the medication. Treatment adherence with sotagliflozin has been facilitated by its favorable dosing schedule and generally manageable side effect profile. Patient education regarding the drug’s mechanism of action and expected benefits has been important for optimizing treatment adherence and managing expectations. The dual mechanism of action provides patients with comprehensive glucose control that may enhance treatment satisfaction compared to single-mechanism therapies (25).

### Sotagliflozin beyond heart failure

Sotagliflozin has been extensively studied for type 1 diabetes management, representing one of its most comprehensively evaluated indications beyond heart failure. The drug was initially evaluated as adjunct therapy to insulin in type 1 diabetes, where it demonstrated significant improvements in glycemic control. Clinical trials in type 1 diabetes showed that sotagliflozin treatment resulted in improved time in target glucose ranges and reduced hyperglycemic excursions (37). The inTandem clinical program extensively evaluated sotagliflozin in adults with type 1 diabetes, demonstrating that the drug could reduce HbA1c levels while providing additional benefits such as weight reduction. Studies showed that sotagliflozin added to optimized insulin therapy led to lower rates of clinically relevant hypoglycemic events at 52 weeks in adults with type 1 diabetes. Despite these clinical benefits, regulatory approval for type 1 diabetes has been limited due to safety concerns (38).

The SCORED trial established sotagliflozin’s role in managing patients with type 2 diabetes and chronic kidney disease, demonstrating important renal and cardiovascular benefits in this population. The trial enrolled patients with moderate renal impairment and demonstrated that sotagliflozin could provide cardiovascular protection in patients with established chronic kidney disease. The drug’s effects on kidney outcomes, kidney function, and albuminuria have been systematically evaluated in this patient population. Sotagliflozin’s dual SGLT1/2 inhibition may provide unique benefits in chronic kidney disease patients by targeting both renal and intestinal glucose handling mechanisms. The drug’s 20:1 SGLT2:SGLT1 receptor affinity provides effective renal glucose handling while offering additional metabolic benefits through intestinal SGLT1 inhibition (23).

Research into sotagliflozin’s applications beyond its current approved indications continues to expand, with investigations into its effects across various cardiovascular and metabolic conditions (39). Studies have suggested potential benefits for stroke prevention in diabetes, with sotagliflozin demonstrating significant stroke risk reduction in clinical trials (40). The presence of SGLT1 in the brain and its overexpression in areas of brain damage could contribute to ischemia and reperfusion injury, supporting sotagliflozin’s unique role in stroke prevention (39). Network meta-analyses have shown that sotagliflozin was one of the most effective drugs for lowering myocardial infarction, stroke, major adverse cardiovascular events, and heart failure hospitalizations. These findings suggest potential applications beyond current approved indications, though additional clinical trials would be needed to establish efficacy and safety in these expanded patient populations (41).

While the SCORED trial’s primary endpoint was cardiovascular, its design in a population with moderate chronic kidney disease (CKD) provided critical insights into sotagliflozin’s reno-protective potential (23). The significant reduction in cardiovascular death and heart failure events in this CKD cohort underscores its safety and efficacy in protecting a vulnerable organ system. Although not a primary renal outcome trial, the mechanism suggests a favorable impact on slowing CKD progression by addressing both the hemodynamic and metabolic drivers of renal injury. This positions sotagliflozin as a compelling therapeutic option for patients with type 2 diabetes and CKD who are at high risk for both cardiovascular and renal events (23).

### International guidelines and recommendations involving sotagliflozin

Sotagliflozin’s integration into international clinical practice guidelines has been evolving as evidence from major clinical trials has become available and regulatory approvals have been obtained. Clinical practice guidelines have begun to recommend dapagliflozin or empagliflozin in all patients with heart failure with reduced ejection fraction, or sotagliflozin in those with heart failure with reduced ejection fraction and concomitant diabetes. The timing and practical integration of these drugs in clinical practice is being refined as experience with their use expands SGLT2 inhibitors, including sotagliflozin, have been recommended as foundational therapy for patients with heart failure and reduced ejection fraction because of their favorable effects on mortality, clinical events, and quality of life. Guidelines are proposing that these drugs are candidates for early, upfront administration to patients with newly diagnosed heart failure with reduced ejection fraction (42).

Physicians’ considerations and practice recommendations regarding the use of sodium-glucose cotransporter-2 inhibitors, including sotagliflozin, have been developed to overcome clinical inertia and address safety concerns. Roundtable discussions with physicians from cardiology, endocrinology, and nephrology specialties have provided practical guidance for sotagliflozin integration into clinical practice. These recommendations address safety considerations that may represent barriers to sotagliflozin use (43). The UK Kidney Association Clinical Practice Guideline for Sodium-Glucose Co-transporter-2 (SGLT-2) Inhibition in Adults with Kidney Disease has provided comprehensive recommendations for SGLT2 inhibitor use, including consideration of sotagliflozin in specific patient populations. These guidelines consider the use of SGLT-2 inhibitors in people with specific medical conditions, including type 1 diabetes, kidney transplants, and people admitted to hospital with heart failure (44).

The development of future guidelines for sotagliflozin will likely incorporate emerging evidence from ongoing clinical trials and real-world experience with the drug. Guidelines for the management of patients with diabetes have become an important part of clinical practice that improve the quality of care and help establish evidence-based medicine. With rapidly accumulating evidence on various aspects of diabetes and cardiovascular care, including landmark clinical trials of treatment agents like sotagliflozin, timely updates of guidelines capture the most current state of the field. Leading academic societies are expected to continue publishing updated practice guidelines that will incorporate growing evidence for sotagliflozin across its various indications. The integration of sotagliflozin into standard treatment algorithms will likely expand as more evidence becomes available and as clinical experience with the drug grows (45).

Sotagliflozin’s unique profile is poised to influence future clinical guidelines significantly (22). As real-world evidence accumulates, guidelines may evolve to specifically recommend dual SGLT1/2 inhibitors for certain patient phenotypes. For instance, patients with heart failure and concomitant poorly controlled postprandial glucose, or those with combined cardiorenal disease and significant gastrointestinal glucose absorption issues, might derive superior benefit from sotagliflozin. Future guideline committees will likely consider the robust, ejection fraction data from SOLOIST-WHF to strengthen recommendations for in-hospital initiation of SGLT inhibitor therapy, with sotagliflozin being a prime candidate due to its trial design (22, 45).

### Future directions and challenges

The landscape of heart failure management is rapidly evolving as advances in molecular biology, precision medicine, and biotechnology offer the promise of transformative change in patient care (6). Despite major advances in pharmacological and device-based therapies, heart failure continues to impose a substantial global health burden with high morbidity and mortality rates, and significant healthcare resource utilization. Emerging approaches focus on harnessing innovations in genomics, proteomics, and metabolomics to identify distinct heart failure phenotypes and underlying molecular mechanisms that can be targeted with precision therapies (46).

Innovative molecular targets, such as microRNAs, epigenetic modulators, cardiac fibroblast signaling hubs, and proteins involved in calcium handling, are gaining traction as potential avenues for new medication development, particularly for subgroups like heart failure with preserved ejection fraction and those with high fibrosis burden (47). Promising developments in cell-based therapies, biologicals, and tissue engineering raise the possibility of myocardial repair or even regeneration, but clinical translation is impeded by challenges related to immune rejection, graft integration, long-term viability, and prohibitive cost---barriers that are especially pronounced in resource-limited settings (46). Patient adherence, healthcare access, coordinated multidisciplinary interventions, and clinician education represent additional real-world obstacles to closing the gap between research breakthroughs and clinical outcomes (6).

The future of heart failure management will rely on integrated, multidimensional strategies that bridge basic science and clinical implementation, including ongoing discovery of new molecular targets, optimization of big data analytics, advances in remote monitoring and telehealth, and a global effort to make emerging innovations accessible and equitable for all affected individuals (46, 48).

Future clinical trials are essential to fully delineate the niche for dual SGLT1/2 inhibition. Key areas for investigation include (47):

- Head-to-head trials against selective SGLT2 inhibitors in populations with predominant postprandial hyperglycemia or advanced CKD to test for superior efficacy.
- Dedicated renal outcome trials in non-diabetic chronic kidney disease, leveraging the dual mechanism’s potential to reduce proteinuria and preserve eGFR.
- Trials exploring combination therapies, for example, with GLP-1 receptor agonists, to exploit the synergistic potential of complementary incretin and glucose-lowering pathways for maximal cardiorenal protection.

## Conclusion

Sotagliflozin, a first-in-class dual SGLT1/2 inhibitor, represents a significant advance in heart failure management by concurrently targeting intestinal and renal glucose pathways. It provides robust cardiovascular and metabolic benefits irrespective of diabetes status or ejection fraction, with a manageable safety profile requiring vigilance for ketoacidosis and gastrointestinal effects. While its adoption trails behind earlier SGLT2 inhibitors due to lesser real-world familiarity and GI tolerability concerns, sotagliflozin offers a distinct mechanistic profile—particularly beneficial in patients with prominent postprandial hyperglycemia or combined cardiorenal disease. Further comparative effectiveness research and long-term outcome studies are needed to fully define its optimal role in clinical practice. Nevertheless, sotagliflozin stands as a potent therapeutic option, enhancing our ability to improve outcomes in heart failure and related cardiometabolic conditions.
